# The effect of smoking, obesity and diabetes on recurrence‐free and overall survival in patients with stage III colon cancer receiving adjuvant chemotherapy

**DOI:** 10.1002/cnr2.1346

**Published:** 2021-02-07

**Authors:** Alex Croese, Richard Gartrell, Richard Hiscock, Margaret Lee, Peter Gibbs, Ian Faragher, Justin Yeung

**Affiliations:** ^1^ Department of Surgery Footscray Hospital Footscray Victoria Australia; ^2^ Melbourne Medical School – Western Health Faculty of Medicine, Dentistry and Health Sciences The University of Melbourne St Albans Victoria Australia; ^3^ Department of Surgery Sunshine Hospital St Albans Victoria Australia; ^4^ Specialist Anesthetist Department of Anesthesia Mercy Hospital for Women Heidelberg Victoria Australia; ^5^ Department of Medical Oncology Eastern Health Box Hill Victoria Australia; ^6^ Department of Medical Oncology Western Health Footscray Victoria Australia; ^7^ Faculty of Medicine, Nursing and Health Sciences Monash University Eastern Health Clinical School Melbourne Victoria Australia; ^8^ Laboratory Head Walter and Eliza Hall Institute Medical Research Parkville Victoria Australia; ^9^ Western Health Head of Colorectal Unit Western Health Footscray Victoria Australia; ^10^ Colorectal Surgical Department Western Health Footscray Victoria Australia; ^11^ Australia Head of Department of Surgery, Melbourne Medical School – Western Health Faculty of Medicine, Dentistry and Health Sciences The University of Melbourne Parkville Victoria Australia

**Keywords:** adjuvant chemotherapy, smoking diabetes obesity Colon cancer

## Abstract

**Background:**

The association between smoking, diabetes and obesity and oncological outcomes in patients with stage III colon cancer treated with surgery and adjuvant chemotherapy is unclear.

**Aim:**

To evaluate whether smoking, obesity and diabetes are associated with the disease‐free survival and overall survival rates of patients with stage III colon cancer who have received adjuvant chemotherapy.

**Methods:**

Patients were selected from the prospectively maintained Australian Cancer Outcomes and Research Database (ACCORD). All stage III colon cancer patients who received adjuvant chemotherapy between January 2003 to December 2015 were retrospectively analyzed. The three primary exposures of interest were smoking status, body mass index (BMI) and diabetic (DM) status. The primary outcomes of interest were disease‐free survival (DFS) and overall survival (OS).

**Results:**

A total of 785 patients between 2003 and 2015 were included for analysis. Using Kaplan‐Meier survivorship curves, there was no association between OS and smoking (*P* = .71), BMI (*P* = .3) or DM (*P* = .72). Similarly, DFS did not reveal an association with smoking (*P* = .34), BMI (*P* = .2) and DM (*P* = .34). Controlling for other covariates the results did not reach statistical significance in adjusted multiple regression models.

**Conclusion:**

Smoking, obesity and DM were not shown to influence DFS or OS for patients with stage III colon cancer who have received adjuvant chemotherapy.

## 1 INTRODUCTION

Colorectal cancer (CRC) is the third most commonly diagnosed cancer in Australia and the second highest cause of cancer death.^1^ Over two‐thirds of patients diagnosed with CRC will have a colonic primary, and of these, approximately 24% will have locoregional nodal involvement [American Joint Committee on Cancer (AJCC) stage III] at diagnosis.^2^, ^3^ Over the past three decades, there has been a significant improvement in the survival rates for patients diagnosed with stage III colon cancer, owing largely to the development of effective adjuvant systemic treatments. Earlier trials demonstrated a survival advantage with adjuvant fluorouracil (5‐FU) and levamisole over observation alone.^4^ More recently, the addition of oxaliplatin to a fluoropyrimidine has been shown to offer further survival benefit and become accepted as a standard of care.^5^ Currently, the 5‐year survival rate for patients with stage III colon cancer is estimated to be 71%.^6^

Smoking, obesity and diabetes mellitus (DM) are patient‐related factors that are associated with an increased risk of developing CRC.^7^, ^8^, ^9^ It has been suggested that smoking may increase the risk of colon cancer by the induction of tumor angiogenesis as well as by the inhibition of apoptosis.^10^ Contrastingly, obesity and DM may promote carcinogenesis through chronic systemic inflammation, hyperinsulinemia and increased levels of circulating insulin‐like growth factors.^8^, ^9^ Therefore, it is not unreasonable to consider the impact of these factors on the disease‐free (DFS) and overall survival (OS) of patients who have been treated for colon cancer. Furthermore, there is some evidence suggesting that smoking, obesity and DM may reduce the efficacy of chemotherapeutic agents.^11^, ^12^, ^13^ Nicotine has been found to decrease the antiproliferative and pro‐apoptotic effects of 5‐FU in in vitro CRC cells, while hyperglycemia has been associated with the diminished efficacy of a number of chemotherapy agents in animal models.^11^, ^13^ Furthermore, visceral obesity may adversely influence pharmacokinetics and drug‐volume distribution resulting in potentiating side effects and subtherapeutically dosing systemic treatments.^12^

There have been several studies that have investigated the impact of patient‐related factors on DFS and OS; however, the majority of these draw data from heterogeneous cohorts with respect to both tumor site (colon and rectal cancers) and disease stage.^14^, ^15^, ^16^ Studies evaluating smoking and survival for stage III colon cancer patients receiving chemotherapy have shown mixed results and may lack broader generalizability to other populations from around the world.^17^, ^18^

The aim of this retrospective Australian cohort study is to determine whether smoking, obesity and DM are associated with DFS and OS rates of patients with stage III colon cancer who have received adjuvant chemotherapy.

## 2 METHODS

A retrospective study was performed identifying patients diagnosed and treated for stage III colon cancer in the Australian Comprehensive Cancer Outcomes and Research Database (ACCORD). The database provides reliable longitudinal patient information across multiple patient‐clinician encounters during their treatment course. Data management and access is provided by BioGrid Australia. Ethical approval for this study (Project ID: 201903/2) was granted by Melbourne Health Human Research Ethics Committee in May 2019. Patients diagnosed with AJCC (eighth ed.) stage III colon cancer between January 2003 and December 2015 were included in the study that encompassed three major colorectal cancer care centers in Melbourne (Footscray Hospital, Royal Melbourne Hospital and Eastern Health). Primary colon tumors were defined as primary adenocarcinomas located from the cecum to the rectosigmoid. Inclusion criteria were patients with stage III colon cancer having undergone curative surgery that received adjuvant chemotherapy. Exclusion criteria included a history of neoadjuvant treatment, no adjuvant systemic therapy or adjuvant radiotherapy. Patient demographic details, cancer diagnosis date, cancer location, surgery type and date, cancer stage, adjuvant treatment, recurrence and survival data were collected. Using the Index of Relative Socio‐Economic Disadvantage (IRSD) score based on patient post code, each patient's ISRD score^1^, ^2^, ^3^, ^4^, ^5^, ^6^, ^7^, ^8^, ^9^, ^10^ was generated. A lower IRSD score indicates a greater level of disadvantage. The Eastern Cooperation Oncology Group (ECOG) performance score was recorded as a measure of patient functional status. The three exposures of interest were smoking status, body mass index (BMI) and diabetic status. Smoking status was defined in the ACCORD registry as either current smoker, ex‐smoker or never smoked. Preoperative height and weight were used to calculate BMI. Patients were categorized as underweight (BMI <18.5), normal weight (18.5‐24.9), overweight (25‐29.9) or obese (>30) based on BMI ranges described by the World Health Organization. Diabetic status was classified as present or absent irrespective of type. OS was defined as time of diagnosis to death and DFS was the time from surgery to recurrence at any site. The primary outcome was the association between smoking status, BMI or DM on DFS and OS in patients with stage III colon cancer.

### 2.1 Statistical analysis

Patient characteristics by overall survival status were summarized as mean (SD), median (minimum, maximum) or number (%) according to type and distribution. Quality of the survival cohort was assessed using the Clarke index, a ratio measure of the sum of observed observation time/potential observed time, based upon overall survival.^19^ BMI was modeled both as a continuous exposure from a referent level of 20 kg.m^−2^ and as a binary exposure cut at BMI 30 kg.m^−2^. Smoking status was modeled as both a three‐level nominal exposure and a binary exposure (currently smoking / not currently smoking). For each outcome (OS & DFS), we present^1^: unadjusted Kaplan‐Meier survivorship curves and associated log‐rank test (categorical variables only)^2^; multivariable Cox proportional hazard (CPH) regression model including age, gender, ECOG, IRSD and oxaliplatin use and the three exposures (BMI, smoking status and DM) and Reference 3. Inverse Probability Weighted (IPW) Cox proportional hazard propensity score modeling of binary exposures to achieve baseline covariate balance. Inverse probability weights were based on the conditional probability of each individual's exposure status calculated using a multivariable logistic model with robust standard errors that included all other covariates including the other two exposures. Covariate balance post weighting was assessed, and weights were trimmed to ensure that the probability distributions for each exposure level overlapped (positivity assumption was met). For all CPH models, the assumption of proportional hazards was assessed using numerical and graphical measures.

Finally, restricted mean survival time (RMST) at 2 and 5 years was calculated using an inverse probability weighted Royston‐Parmar restricted cubic spline model. This provides a robust measure of mean survival time, even if the proportional hazards assumption is not met, and an expected difference in mean survival time between exposure groups.

We did not undertake imputation of missing covariate values and regression results are presented using point estimates and associated 95% confidence intervals. Statistical software used was Stata Release 16 (StataCorp. 2019. *Stata Statistical Software: Release 16*. College Station, TX: StataCorp LLC) and RMST estimation used the strmst program.^20^

## 3 RESULTS

### 3.1 Patient Characteristics

A total of 785 patients, who underwent surgery between January 1, 2003 and January 20, 2015, were included in the analysis. The date of last follow‐up was the June 6, 2019. Based upon overall survival the Clarke index, the mean ratio of actual to potential follow‐up time was 64.7%. Baseline characteristics for the entire cohort are listed in Table 1 and further subdivided according to primary exposure in Table 2. Four hundred and seventeen (53.1%) were male with an average age of 64 years. Three hundred and fifty‐nine (46%) patients had a high IRSD score between 8 and 10 indicating relatively low socioeconomic disadvantage. Five hundred and ninety‐one (75.3%) patients had an ECOG status of ≤2. Three hundred and ninety‐seven patients (50.6%) had left‐colonic primaries (distal to the splenic flexure). Sixty‐one (43.3%) patients with DM had a right‐sided colon malignancy up to, but not including the splenic flexure. Of patients with complete information, 669 (87.5%) patients had T3/4 disease and 602 patients (77.3%) had N1 disease. Two hundred and ninety‐two (39.4%) patients were either ex or current smokers, 179 patients (28.2%) were classified as obese with a BMI ≥30 and 141 (18.5%) were diabetic. At 5‐years of follow‐up, unadjusted DFS was 72% (95% CI 68‐75), and OS was 79% (95% CI 76‐82).

**TABLE 1:** Baseline patient characteristics

	Alive	Deceased	Total
	N = 603	N = 182	N = 785
Patient characteristics			
Age (y)			
mean (SD)	64 (12.1)	66 (11.6)	64 (12.0)
median (minimum, maximum)	65.0 (14.9, 87.8)	66.9 (25.4, 88.0)	65.3 (14.9, 88.0)
Gender (Male)	311 (51.6%)	106 (58.2%)	417 (53.1%)
Smoking status			
Never	354 (61.8%)	96 (56.8%)	450 (60.6%)
Ex	153 (26.7%)	60 (35.5%)	213 (28.7%)
Current	66 (11.5%)	13 (7.7%)	79 (10.6%)
Missing data			43 (5.5%)
Body mass (kg.m^−2^)			
mean (SD)	28 (5.5)	27 (5.2)	28 (5.4)
Body mass index (6 level)			
<18.5	4 (0.8%)	1 (0.7%)	5 (0.8%)
≥18.5, <25	157 (31.9%)	51 (35.9%)	208 (32.8%)
≥25, <30	186 (37.8%)	56 (39.4%)	242 (38.2%)
≥30, <35	94 (19.1%)	23 (16.2%)	117 (18.5%)
≥35, <40	36 (7.3%)	8 (5.6%)	44 (6.9%)
≥40	15 (3.0%)	3 (2.1%)	18 (2.8%)
Body mass index ≥30 kgm^−2^ (Yes)	145 (29.5%)	34 (23.9%)	179 (28.2%)
Missing data			151 (19.2%)
Diabetes			
No	486 (80.6%)	134 (73.6%)	620 (79%)
Yes	102 (16.9%)	39 (21.4%)	141 (18%)
Missing data	15 (2.5%)	9 (5.0%)	24 (3.0%)
ECOG			
0‐2	467 (77.4%)	124 (68.1%)	591 (75.3%)
≥3	136 (22.6%)	58 (31.9%)	194 (24.7%)
IRSD			
1‐4	104 (17.3%)	43 (23.6%)	147 (18.8%)
5‐7	211 (35.2%)	65 (35.7%)	276 (35.3%)
8‐10	285 (47.5%)	74 (40.7%)	359 (45.9%)
Tumor characteristics			
Tumor stage			
1‐2	90 (15.2%)	6 (3.5%)	96 (12.5%)
3‐4	500 (84.8%)	169 (96.5%)	669 (87.5%)
Nodal stage			
1	478 (79.5%)	124 (69.7%)	602 (77.3%)
2	123 (20.5%)	54 (30.3%)	177 (22.7%)
Post‐operative treatment			
Adjuvant therapy regimen			
Single agent	203 (34.5%)	101 (55.8%)	304 (39.5%)
Oxaliplatin Doublet	385 (65.4%)	80 (44.2%)	465 (60.4%)
Other	1 (0.2%)	0 (0.0%)	1 (0.1%)
Recurrence outcome			
Recurrence (Yes)	69 (11.4%)	150 (82.4%)	219 (27.9%)
Local	15 (2.5%)	58 (31.9%)	73 (9.3%)
Distant	58 (9.6%)	118 (64.8%)	176 (22.4%

Abbreviations: ECOG, European Cooperative Oncology Group; IRSD, Index of Relative Socio‐economic Disadvantage; SD, Standard deviation.

**TABLE 2:** Baseline characteristics according to exposure

	Currently smoking		diabetes mellitus		BMI >30	
	No	Yes	No	Yes	No	Yes
	n = 663	n = 79	n = 620	n = 141	n = 455	n = 179
Missing	n = 43		n = 24		n = 151	
Patient characteristics						
Age (y)						
mean (SD)	65.0 (12.0)	58.7 (11.7)	63.4 (12.4)	68.0 (9.6)	64.7 (12.1)	63.3 (11.0)
Median (minimum, maximum)	65.6 (14.9,88.0)	58.6 (25.4,81.0)	64.4 (14.9,87.8)	68.8 (40.1,88.0)	65.4 (23.3,88.0)	64.9 (29.5,87.8)
Gender (Male)	345 (52.0%)	46 (58.2%)	323 (52.1%)	82 (58.2%)	259 (56.9%)	79 (44.1%)
Smoking status (Yes)	‐	‐	66 (11.0%)	13 (9.6%)	62 (14.1%)	15 (8.4%)
BMI ≥30 kgm^−2^ (Yes)	163 (30.1%)	15 (19.5%)	120 (24.0%)	58 (45.0%)	‐	‐
Diabetes (Yes)	123 (18.7%)	13 (16.5%)	‐	‐	71 (15.7%)	58 (32.6%)
ECOG						
≤2	488 (73.6)	65 (82.3%)	481 (77.6%)	88 (62.4%)	356 (78.2%)	128 (71.5%)
IRSD						
1‐4	113 (17.1%)	24 (30.4%)	100 (16.2%)	44 (31.4%)	84 (18.5%)	46 (25.8%)
5‐7	234 (35.4%)	30 (38.0%)	220 (35.6%)	50 (35.7%)	154 (34.0%)	68 (38.2%)
8‐10	314 (47.5%)	25 (31.6%)	298 (48.2%)	46 (32.9%)	215 (47.5%)	64 (36.0%)
Tumor characteristics						
Tumor stage						
1‐2	84 (12.7%)	6 (7.6%)	81 (13.1%)	12 (8.5%)	51 (11.2%)	26 (14.5%)
3‐4	571 (86.1%)	73 (92.4%)	528 (85.2%)	128 (90.8%)	397 (87.3%)	150 (83.8%)
Nodal stage						
1	509 (77.0%)	61 (77.2%)	467 (75.7%)	117 (83.0%)	354 (78.0%)	136 (76.0%)
2	152 (23.0%)	18 (22.8%)	150 (24.3%)	24 (17.0%)	100 (22.0%)	43 (24.0%)
Post‐operative treatment						
Adjuvant therapy regimen						
Single agent	268 (41.1%)	22 (27.8%)	221 (36.4%)	74 (52.9%)	178 (39.5%)	71 (40.1%)
OXALI based	383 (58.7%)	57 (72.2%)	386 (63.6%)	65 (46.4%)	273 (60.5%)	105 (59.3%)
Other	1 (0.2%)	0 (0.0%)	0 (0.0%)	1 (0.7%)	0 (0.0%)	1 (0.6%)
Recurrence outcome						
Recurrence (Yes)	181 (27.3%)	20 (25.3%)	165 (26.6%)	44 (31.2%)	127 (27.9%)	44 (24.6%)
Local	52 (7.8%)	1 (1.3%)	43 (6.9%)	11 (7.8%)	35 (7.7%)	12 (6.7%)
Distant	143 (21.6%)	18 (22.8%)	132 (21.3%)	36 (25.5%)	103 (22.6%)	34 (19.0%)

Abbreviations: ECOG, European Cooperative Oncology Group; IRSD, Index of Relative Socio‐economic Disadvantage; SD, Standard deviation.

### 3.2 Smoking status

**FIGURE 1:**
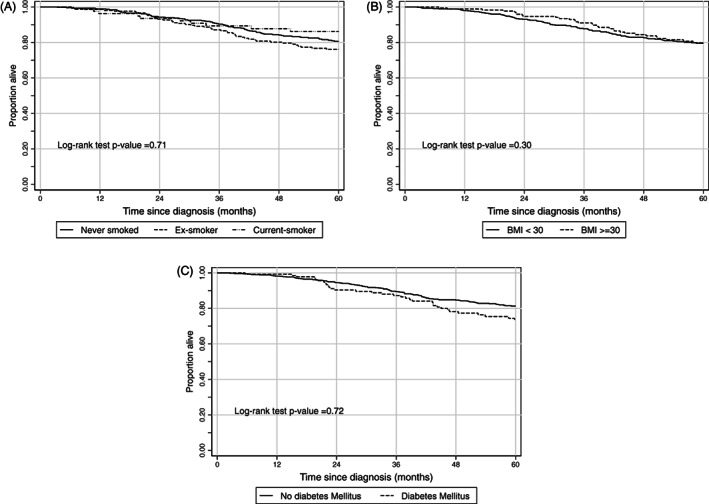
Unadjusted Kaplan‐Meier survival curves for overall survival

Smoking status modeled as a three‐level nominal variable (never, ex and current) was not associated with overall survival when unadjusted (*P* = .71, Figure 1A) or adjusted using a multivariable regression model. As a binary model, there was no difference in mean restricted survival time at 2 or 5 years (Table 3). The proportional hazards assumption was violated in the IPW regression model and survival cannot be appropriately summarized by a single hazard ratio. The overall 5‐year survival for non‐smokers, ex‐smokers and current smokers was 80.6% (95% CI 76.3‐84.2), 76.0% (95% CI 69.3‐81.5) and 86.1% (95% CI 75.7‐92.3 respectively).

**TABLE 3:** Associations of overall and recurrence free survival with smoking status, body mass index and diabetes mellitus

	Log‐rank test *P*‐value	HR (95%CI) Multivariable regression model	HR (95%CI) IPW propensity score model	RMST (95%CI) IPW at 2 y (mo) Referent	RMST (95%CI) IPW at 2 y (mo) Difference	RMST (95%CI) IPW at 5 y (mo) Referent	RMST (95%CI) IPW at 5 y (mo) Difference
**Overall survival**							
Smoking status	(n = 742)	(n = 610)	(n = 591)				
Never	.71	referent	‐	‐	‐	‐	‐
Ex		1.04 (0.71 to1.53)	‐	‐	‐	‐	‐
Currently smoking (yes)	.45	0.67 (0.37 to 1.23)	0.35 (0.15 to 0.84)^a^	23.6 (23.4 to 23.8)	0.1 (−0.2 to 0.5)	53.9 (51.8to 56.0)	3.2 (−0.5 to 5.8)
Body mass index	(n = 634)	(n = 610)	(n = 609)				
BMI (5‐unit change kgm^−2^)	‐	0.85 (0.71 to 1.01)	‐	‐	‐	‐	‐
BMI ≥30 kgm^−2^	.30	0.66 (0.44 to 1.02)	0.67 (0.44 to 1.03)	23.5 (23.3 to 23.8)	0.1 (−0.3 to 0.4)	54.1 (52.8 to 55.5)	1.4 (−1.0 to 3.8)
Diabetes mellitus	(n = 761)	(n = 610)	(n = 571)				
Yes	.72	1.19 (0.77 to 1.84)	1.29 (0.82 to 2.02)	23.6 (23.3 to 23.8)	−0.1 (−0.5 to 0.3)	54.6 (53.4 to 55.8)	−1.2 (−4.6 to 2.2)
**Recurrence free survival**							
Smoking status	(n = 741)	(n = 609)	(n = 590)				
Never	.34	referent	‐	‐	‐	‐	‐
Ex		1.13 (0.80 to 1.60)^a^	‐	‐	‐	‐	‐
Current		0.95 (057 to 1.58)^a^	‐	‐	‐	‐	‐
Currently smoking (yes)	.29	0.91 (0.56 to 1.49)^a^	1.25 (0.53‐2.93)	21.6 (20.7 to 22.6)	−0.6 (−3.2 to 2.1)	47.9 (44.2to 51.5)	−2.5 (−13.2 to 8.2)
Body mass index	(n = 632)	(n = 609)	(n = 608)				
BMI (5‐unit change kgm^−2^)	‐	0.98 (0.84 to 1.13)^a^	‐	‐	‐	‐	‐
BMI ≥30 kgm^−2^	.20	0.80 (0.56 to 1.16)^a^	0.81 (0.56 to 1.19)	21.8 (21.3 to 22.3)	0.3 (−0.6 to 1.1)	48.5 (46.6 to 50.4)	1.7 (−2.0 to 5.3)
Diabetes mellitus	(n = 758)		(n = 570)				
Yes	.34	1.15 (0.78 to 1.71)^a^	1.23 (0.80 to 1.89)	22.0 (21.5 to 22.5)	−0.4 (−1.3 to 0.47)	48.7 (46.9 to 50.6)	−2.2 (−6.9 to 2.4)

Abbreviations: HR, hazard ratio; IPW, inverse probability weighted; RMST, restricted mean survival time; 95%CI, 95% Confidence Interval.

^a^ Proportional Hazards assumption not met, use of single HR to summarize survival is not appropriate.

**FIGURE 2:**
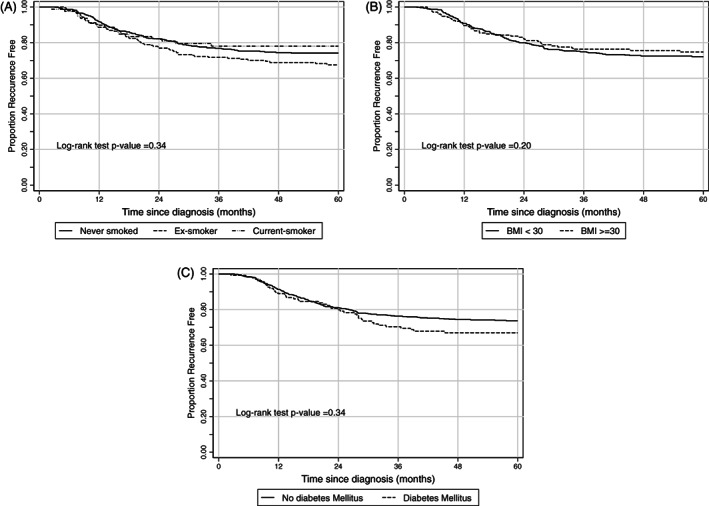
Unadjusted Kaplan‐Meier survival curves for disease free survival

The 5‐year DFS for non, ex and current smokers was 74.12% (95% 69.7‐78.1), 67.5% (60.4‐73.6) and 78% (67.0‐85.7), respectively. There was no evidence for a DFS difference by smoking status. The Kaplan‐Meier survivorship curve and log rank test (*P* = .34) are presented in Figure 2A. For both multivariable and IPW Cox regression modeling, the PH assumption was violated and the use of a constant hazard ratio across follow‐up time is not appropriate. Based upon RMST, there is no evidence for a difference in DFS between those currently and not currently smoking (Table 3).

### 3.3 Body mass index

There was no evidence for a difference in survival with increasing BMI. The Kaplan‐Meier survivorship curve and log rank test (*P* = .30) are presented in Figure 1B. Unadjusted the 5‐year survival for patients with a BMI <30 and ≥30 was 79.5% (95% CI 75.2‐83.14) and 79.8% (95% CI 72.4‐85.4) respectively. Cox proportional hazards models for continuous BMI, BMI cut at 30 kgm^−2^ and IPW all indicate no association of BMI and overall survival. The proportional hazards (PH) assumption was met in all models. There was no difference in RMST between exposure levels at either 2 or 5 years (Table 3).

The 5‐year DFS for BMI <30 and ≥ 30 was 72.1% (95% CI 67.6‐76.2) and 74.7% (67.3‐80.7). There was no evidence for a DFS difference by BMI. The Kaplan‐Meier survivorship curve and log rank test (*P* = 0.20) are presented in Figure 2B. The PH assumption was violated for multivariable Cox regression model. Both the IPW hazard ratio and RMST indicate no evidence for an association between BMI and DFS (Table 3).

### 3.4 Diabetes mellitus

The unadjusted 5‐year survival was 79.8% (95% CI 72.4‐85.4) and 81.3% (95 CI 77.7‐84.3) for diabetics and non‐diabetics, respectively. There was no evidence for a survival difference by DM status. The Kaplan‐Meier survivorship curve and log rank test (*P* = 0.72) are presented in Figure 1C. The multivariable Cox proportional hazards model and the IPW mode both indicated no association between DM and overall survival. The PH assumption was met in all models. There was no difference in RMST between exposure levels at either 2 or 5 years (Table 3).

The 5‐year DFS for diabetic and non‐diabetic patients was 73.7% (95% 70.0‐77.2) and 67.0% (67.3‐80.7). There was no evidence for a DFS difference by DM status. The Kaplan‐Meier survivorship curve and log rank test (*P* = .34) are presented in Figure 2C. The PH assumption was violated for the multivariable regression model. The IPW hazard ratio and RMST indicate no evidence for an association between BMI and DFS (Table 3).

## 4 DISCUSSION

We found no evidence for an association between smoking, obesity or DM with either DFS or OS on our Australian cohort of patients with stage III colon cancer who underwent adjuvant chemotherapy.

With respect to smoking status, our findings are both consistent and contradictory with current published literature.^17^, ^18^ A number of similar studies have also been published but use heterogenous cohorts.^21^, ^22^, ^23^ McCleary et al^17^ investigated the effects of smoking on survival for stage III colon patients using data from a randomized control trial (RCT) evaluating the role of adjuvant irinotecan and 5‐FU/leucovorin. They found no association with either DFS or OS.^17^ This study did however find that subjects with a 20+ pack year history of smoking, compared to non‐smokers, were associated with an unadjusted hazard ratio (HR) of 1.34 (95% CI, 1.02‐1.75) for cancer recurrence or death from any cause. The dose‐dependent relationship associated with smoking appeared to be nonsignificant in multivariant analysis for all other variables. In another study, which also used data from a multicenter RCT investigating adjuvant chemotherapy regimens, a history of smoking was shown to have a statistically significant association with poorer DFS and OS in patients with stage III colon cancer.^18^ This study found that current smokers compared to former smokers had poorer DFS outcomes compared to never smokers (HR 1.47 95% CI, 1.04‐2.09). Unlike McCleary et al, this study did not show a significant dose‐dependent relationship. There have been several suggested mechanisms of how smoking may negatively impact upon patients being treated for CRC. First, current smokers have been shown to have a significantly increased risk of perioperative morbidity and mortality.^24^ Smoking has also been found to reduce the efficacy of systemic treatments such as 5‐FU and cetuximab, as well as promoting colon cancer cell migration.^11^, ^25^, ^26^ Despite the above, there is no convincing clinical evidence that smoking influences DFS and OS in stage III colon cancer patients receiving chemotherapy. The significance of smoking status on outcomes may be confounded by the fact that patients who receive chemotherapy must have a relatively robust baseline level of physical health. This fact may have resulted in a selection bias, potentially reflected by the finding that the majority of patients had an ECOG score of ≤2. Furthermore, the results of the current study showed a nonsignificant trend toward improved survival for current smokers (HR 0.65, 95% CI 0.37‐1.12) that remained consistent across univariant and multivariant analysis. Another consideration is that a history of smoking has been found to be associated with an increased risk of developing tumors with high levels of microsatellite instability (MSI‐H).^27^ Patients with MSI‐H tumors have also been shown to have better prognosis with better DFS and OS when compared to patients with microsatellite stable (MSS) tumors.^28^ Therefore, it is conceivable that cohorts of patients with colon cancer who are smokers will have a greater percentage of biologically favorable tumors, giving an apparent overall survival advantage to the cohort. There is however some evidence that suggests smoking may have a negative impact on survival outcomes for patients with MSI‐H tumors, although this interaction was limited to tumors that were *BRAF* wild type.^28^ The above conflicting theories and current evidence suggest that further research is required to better define the potential interaction between smoking and survival outcomes for patients with MSI‐H tumors.

To the author's knowledge, only one other recent study assessing the impact of obesity on stage III colon cancer survival in patients receiving chemotherapy has been published^29^ with the authors also finding no association between BMI and survival outcomes. Much of the available remaining evidence related to obesity and survival in patients with CRC is largely based on heterogenous cohorts which include patients with both rectal and colonic malignancies across multiple stages of disease. Alexander et al showed that overweight (BMI 25‐29.9) and obese (BMI >30) patients had a higher risk of all‐cause mortality compared to normal weight patients with a HR of 2.81 (95% CI, 1.24, 6.35).^29^ Boyle et al investigated the impact of obesity on CRC‐specific mortality and found that overweight, but not obese patients, had a statistically significant increased risk of CRC cancer‐specific mortality (HR 1.51, 95% CI = 1.04, 2.18).^14^ In direct contrast, Daniel et al, who found a U‐shaped relationship between BMI and survival in CRC patients.^15^ Specifically, they found that patients with a BMI of 25‐29.9 had the lowest risk of death. The conflicting literature may be reflective of BMI not being the optimal way to assess obesity or body fat composition, and therefore an individual's risk of health‐related consequences of obesity.^30^ This has been highlighted in two studies which have utilized waist circumference and visceral fat area (VFA), respectively, to assess obesity.^12^, ^16^ Both found that their respective measure of obesity had a statistically significant association with DFS and OS, while neither of the studies could identify a significant association with BMI.

Our finding that DM has no association with DFS, or OS is in keeping with the results of two other cohort studies.^31^, ^32^ Although these studies only included colon cancer patients, they did however include patients with a range of cancer stages. In a meta‐analysis investigating the survival outcomes for patients with CRC, Mills et al identified a statistically significant association between DM and reduced survival for both rectal and colon cancer patients; however, they did not find an association with recurrence for either cancer type.^33^ The authors suggested that less aggressive treatment of diabetic patients as well as a potentially reduced response to chemotherapy may have led to this reduction in survival. Determining the true impact of diabetes on colon cancer survival and recurrence is complex as much of the data is retrospective and lacks objective markers of disease severity and levels of hyperglycemia, such as glycosylated hemoglobin levels (HbA1c). There is also some evidence showing that treatment with metformin may offer a protective effect and reduce CRC‐specific deaths. It has been suggested that metformin reduces the circulating levels of insulin like growth factors, as well as the synthesis of certain proteins that are key in the production of malignant cancer cells and angiogenesis.^33^ Therefore, documentation and analysis of diabetic treatment regimens of study cohorts may need to be examined closely to assess whether these variables may impact on the survival outcomes.

This study is limited by its retrospective nature and presence of missing data. Imputation techniques to account for missing data were not performed due to their inherent problems in adequately accounting for selection bias. This study is also limited by the lack of objective markers of disease severity of diabetes such as glycosylated hemoglobin, and exposure severity of smoking such as a pack year history. Our study's strength is the size of cohort and the homogenous cohort with the inclusion of only patients with stage III colon cancer who received postoperative chemotherapy.

## 5 CONCLUSION

In our Australian cohort of patients with stage III colon cancer who received adjuvant chemotherapy, there was no association between smoking, obesity or DM and DFS or OS outcomes.

## CONFLICT OF INTEREST

The authors have stated explicitly that there are no conflicts of interest in connection with this article.

## AUTHOR CONTRIBUTIONS

Conceptualization; data curation; formal analysis; funding acquisition; investigation; methodology; project administration; writing‐original draft, A.C.; Data curation; formal analysis; investigation; methodology; project administration; writing‐review and editing, R.G.; Formal analysis; methodology; software; supervision, R.H.; Writing‐review and editing, M.L.; Writing‐review and editing, P.G.; Conceptualization; methodology; writing‐review and editing, I.F.; Conceptualization; methodology; project administration; supervision; writing‐review and editing, J.Y.

## ETHICAL STATEMENT

Ethical approval for this study (Project ID: 201903/2) was granted by Melbourne health Human Research Ethics Committee in May 2019.

## PATIENT CONSENT STATEMENT

This was a retrospective audit involving access to existing medical records and the research personnel reviewing these records would normally have access to these records. Therefore, patient consent was waivered.

## Data Availability

The data that support the findings of this study are available on request from the corresponding author. The data are not publicly available due to privacy or ethical restrictions. All de‐identified data accessed from the ACCORD database has been confidentially stored and is accessible upon reasonable request to the principle author.

## References

[1] Bowel Cancer Australia . Bowel Cancer Facts & Information 2019. https://www.bowelcanceraustralia.org/facts. Accessed July 17, 2020

[2] Brouwer NPM , Bos A , Lemmens V , et al. An overview of 25 years of incidence, treatment and outcome of colorectal cancer patients. Int J Cancer. 2018;143(11):2758‐2766. 30095162 10.1002/ijc.31785 PMC6282554

[3] Cancer Australia . Feature: National cancer stage at diagnosis data. Strawberry Hills, NSW: 2018.

[4] Moertel CG , Fleming TR , Macdonald JS , et al. Levamisole and fluorouracil for adjuvant therapy of resected colon carcinoma. N Engl J Med. 1990;322(6):352‐358. 2300087 10.1056/NEJM199002083220602

[5] Andre T , Boni C , Mounedji‐Boudiaf L , et al; Multicenter International Study of Oxaliplatin/5‐Fluorouracil/Leucovorin in the Adjuvant Treatment of Colon Cancer (MOSAIC) Investigators. Oxaliplatin, fluorouracil, and leucovorin as adjuvant treatment for colon cancer. N Engl J Med. 2004;350(23):2343‐2351. 15175436 10.1056/NEJMoa032709

[6] American Society of Clinical Oncology . Colorectal Cancer: Statistics. 2020. https://www.cancer.net/cancer‐types/colorectal‐cancer/statistics#:~:text=The%205%2Dyear%20survival%20rate%20of%20people%20with%20localized%20stage,year%20survival%20rate%20is%2071%25. Accessed July 18 2020.

[7] Liang PS , Chen TY , Giovannucci E . Cigarette smoking and colorectal cancer incidence and mortality: systematic review and meta‐analysis. Int J Cancer. 2009;124(10):2406‐2415. 19142968 10.1002/ijc.24191

[8] Dong Y , Zhou J , Zhu Y , et al. Abdominal obesity and colorectal cancer risk: systematic review and meta‐analysis of prospective studies. Biosci Rep. 2017;37(6):1‐12. 10.1042/BSR20170945 PMC5725611 29026008

[9] Yuhara H , Steinmaus C , Cohen SE , Corley DA , Tei Y , Buffler PA . Is diabetes mellitus an independent risk factor for colon cancer and rectal cancer? Am J Gastroenterol. 2011;106(11):1911‐1921; quiz 22. 21912438 10.1038/ajg.2011.301 PMC3741453

[10] O'Byrne KJ , Dalgleish AG , Browning MJ , Steward WP , Harris AL . The relationship between angiogenesis and the immune response in carcinogenesis and the progression of malignant disease. Eur J Cancer. 2000;36(2):151‐169. 10741273 10.1016/s0959-8049(99)00241-5

[11] Dinicola S , Morini V , Coluccia P , et al. Nicotine increases survival in human colon cancer cells treated with chemotherapeutic drugs. Toxicol In Vitro. 2013;27(8):2256‐2263. 24095863 10.1016/j.tiv.2013.09.020

[12] Lee CS , Murphy DJ , McMahon C , et al. Visceral adiposity is a risk factor for poor prognosis in colorectal cancer patients receiving adjuvant chemotherapy. J Gastrointest Cancer. 2015;46(3):243‐250. 25832480 10.1007/s12029-015-9709-0

[13] Gerards MC , van der Velden DL , Baars JW , et al. Impact of hyperglycemia on the efficacy of chemotherapy‐A systematic review of preclinical studies. Crit Rev Oncol Hematol. 2017;113:235‐241. 28427512 10.1016/j.critrevonc.2017.03.007

[14] Boyle T , Fritschi L , Platell C , Heyworth J . Lifestyle factors associated with survival after colorectal cancer diagnosis. Br J Cancer. 2013;109(3):814‐822. 23787918 10.1038/bjc.2013.310 PMC3738138

[15] Daniel CR , Shu X , Ye Y , et al. Severe obesity prior to diagnosis limits survival in colorectal cancer patients evaluated at a large cancer centre. Br J Cancer. 2016;114(1):103‐109. 26679375 10.1038/bjc.2015.424 PMC4716542

[16] Jayasekara H , English DR , Haydon A , et al. Associations of alcohol intake, smoking, physical activity and obesity with survival following colorectal cancer diagnosis by stage, anatomic site and tumor molecular subtype. Int J Cancer. 2018;142(2):238‐250. 28921583 10.1002/ijc.31049

[17] McCleary NJ , Niedzwiecki D , Hollis D , et al. Impact of smoking on patients with stage III colon cancer: results from cancer and leukemia group B 89803. Cancer. 2010;116(4):957‐966. 20052723 10.1002/cncr.24866 PMC2884300

[18] Phipps AI , Shi Q , Newcomb PA , et al. Associations between cigarette smoking status and colon cancer prognosis among participants in North Central Cancer Treatment Group Phase III Trial N0147. J Clin Oncol. 2013;31(16):2016‐2023. 23547084 10.1200/JCO.2012.46.2457 PMC3661936

[19] Clark TG , Altman DG , De Stavola BL . Quantification of the completeness of follow‐up. Lancet. 2002;359(9314):1309‐1310. 11965278 10.1016/s0140-6736(02)08272-7

[20] Royston P . Estimating the treatment effect in a clinical trial using the difference in restricted mean survival time. Stata J. 2015;15:1098‐1117.

[21] Huang YC , Lin JK , Chen WS , et al. Diabetes mellitus negatively impacts survival of patients with colon cancer, particularly in stage II disease. J Cancer Res Clin Oncol. 2011;137(2):211‐220. 20387072 10.1007/s00432-010-0879-7 PMC11828289

[22] Meyerhardt JA , Catalano PJ , Haller DG , et al. Impact of diabetes mellitus on outcomes in patients with colon cancer. J Clin Oncol. 2003;21(3):433‐440. 12560431 10.1200/JCO.2003.07.125

[23] van de Poll‐Franse LV , Haak HR , Coebergh JW , Janssen‐Heijnen ML , Lemmens VE . Disease‐specific mortality among stage I‐III colorectal cancer patients with diabetes: a large population‐based analysis. Diabetologia. 2012;55(8):2163‐2172. 22526616 10.1007/s00125-012-2555-8 PMC3390707

[24] Sharma A , Deeb AP , Iannuzzi JC , Rickles AS , Monson JR , Fleming FJ . Tobacco smoking and postoperative outcomes after colorectal surgery. Ann Surg. 2013;258(2):296‐300. 23059503 10.1097/SLA.0b013e3182708cc5

[25] Wei PL , Kuo LJ , Huang MT , et al. Nicotine enhances colon cancer cell migration by induction of fibronectin. Ann Surg Oncol. 2011;18(6):1782‐1790. 21210228 10.1245/s10434-010-1504-3

[26] Ordonez‐Mena JM , Walter V , Schottker B , et al; Consortium on Health and Ageing: Network of Cohorts in Europe and the United States (CHANCES). Impact of prediagnostic smoking and smoking cessation on colorectal cancer prognosis: a meta‐analysis of individual patient data from cohorts within the CHANCES consortium. Ann Oncol. 2018;29(2):472‐483. 29244072 10.1093/annonc/mdx761 PMC6075220

[27] Slattery ML , Curtin K , Anderson K , et al. Associations between cigarette smoking, lifestyle factors, and microsatellite instability in colon tumors. J Natl Cancer Inst. 2000;92(22):1831‐1836. 11078760 10.1093/jnci/92.22.1831

[28] Guastadisegni C , Colafranceschi M , Ottini L , Dogliotti E . Microsatellite instability as a marker of prognosis and response to therapy: a meta‐analysis of colorectal cancer survival data. Eur J Cancer. 2010;46(15):2788‐2798. 20627535 10.1016/j.ejca.2010.05.009

[29] Alexander D , Allardice GM , Moug SJ , Morrison DS . A retrospective cohort study of the influence of lifestyle factors on the survival of patients undergoing surgery for colorectal cancer. Colorectal Dis. 2017;19(6):544‐550. 28027419 10.1111/codi.13594

[30] Chang SH , Beason TS , Hunleth JM , Colditz GA . A systematic review of body fat distribution and mortality in older people. Maturitas. 2012;72(3):175‐191. 22595204 10.1016/j.maturitas.2012.04.004 PMC3367099

[31] Shonka NA , Anderson JR , Panwalkar AW , Reed EC , Steen PD , Ganti AK . Effect of diabetes mellitus on the epidemiology and outcomes of colon cancer. Med Oncol. 2006;23(4):515‐519. 17303910 10.1385/MO:23:4:515

[32] Chubak J , Yu O , Ziebell RA , et al. Risk of colon cancer recurrence in relation to diabetes. Cancer Causes Control. 2018;29(11):1093‐1103. 30244297 10.1007/s10552-018-1083-3 PMC6230488

[33] Mills KT , Bellows CF , Hoffman AE , Kelly TN , Gagliardi G . Diabetes mellitus and colorectal cancer prognosis: a meta‐analysis. Dis Colon Rectum. 2013;56(11):1304‐1319. 24105007 10.1097/DCR.0b013e3182a479f9 PMC3800045

